# Children’s Dietary Quality and Micronutrient Adequacy by Food Security in the Household and among Household Children

**DOI:** 10.3390/nu11050965

**Published:** 2019-04-27

**Authors:** Shinyoung Jun, Mary J. Zeh, Heather A. Eicher-Miller, Regan L. Bailey

**Affiliations:** 1 Department of Nutrition Science, Purdue University, West Lafayette, IN 47907, USA; jun24@purdue.edu (S.J.); heicherm@purdue.edu (H.A.E.-M.); 2 Human Biology Program, Indiana University Bloomington, Bloomington, IN 47405, USA; zehj@iu.edu

**Keywords:** food insecurity, household food security, food security among household children, dietary quality, Healthy Eating Index, nutrient adequacy, Mean Adequacy Ratio, children, NHANES

## Abstract

Children’s food-security status has been described largely based on either the classification of food security in the household or among household children, but few studies have investigated the relationship between food security among household children and overall dietary quality. Our goal was to examine children’s dietary quality and micronutrient adequacy by food-security classification for the household and among household children. Data from 5540 children (2–17 years) from the National Health and Nutrition Examination Survey (NHANES) 2011–2014 were analyzed. Food-security status was assessed using the U.S. Household Food Security Survey Module and categorized into high, marginal, low, and very low food security for the households and among household children. Dietary quality and micronutrient adequacy were characterized by the Healthy Eating Index (HEI) 2015 and Mean Adequacy Ratio (MAR; based on total nutrient intakes from diet and dietary supplements), respectively. The HEI 2015 scores did not substantially vary by either food-security classification, but the MAR was greater in high compared to very low food security in households and among household children; a linear relationship was found only among household children. In general, very good agreement was observed between the classifications, but the strength of agreement differed by children’s age, race/Hispanic origin, and family income. In conclusion, micronutrient adequacy, but not dietary quality, significantly differed by food-security status. While the agreement between food security in the household and among household children is very good, classification of food security among household children may be more sensitive to detecting differences in exposure to nutrients.

## 1. Introduction

Food insecurity occurs when consistent access to enough food for an active and healthy life is limited or uncertain due to lack of resources for food [1,2]. Food-security status in the United States (U.S.) has been assessed since 1995 by the U.S. Department of Agriculture (USDA) using the U.S. Household Food Security Survey Module (HFSSM) [3]. The USDA classification of food insecurity represents a range of experiences characterizing limited resources for food: high food security is defined as no indication of limits to food access, marginal food security as anxiety about securing food but little indication of changes in diet or food intake, low food security as reduced quality of diet, and very low food security as altered eating patterns and reduced quantity of food intake [2]. Food insecurity is quantified for the entire household using the full set of questions in the HFSSM, adults in the household using the 10 adult-specific items in the HFSSM, and children in the household using the eight child-specific items. The ranges of experience for the household or children in the household (i.e., household children) are classified using the Household Food Security Scale or the Child Food Security Scale, respectively. Such a tailored approach to food-security classification recognizes that the experience of children may be different with adults living in the same household. In 2017, household food insecurity (i.e., food insecurity in the household) was estimated at 15.7% among households with children, 11.6% with low food security and 4.1% with very low food security [2]. Meanwhile, in 7.7% of households with children, at least one child was food-insecure (i.e., food insecurity among household children), suggesting that children may not have directly experienced food insecurity in about half of food-insecure households with children.

Previous systematic and narrative reviews of U.S. studies showed evidence of adverse associations between food insecurity and dietary outcomes among children that may vary by age, although less consistent when compared to adults [4,5]. Among the studies that utilized the HFSSM or its short form, many used the Household Food Security Scale to describe children’s food-security status, and a few studies used the Child Food Security Scale to investigate the relationship with dietary intake [4]. The choice of food-security scale may impact the resulting relationship discovered between food security and dietary intake. Agreement between the scales has not been evaluated since the development of the Child Food Security Scale [6]. Furthermore, most studies focused on individual nutrients and food groups rather than overall dietary quality. Several indices of dietary quality were developed to reflect multiple components of the human diet [7]. The Healthy Eating Index (HEI) measures adherence to the Dietary Guidelines for Americans (DGA), primarily based on food intake [8], whereas the Mean Adequacy Ratio (MAR) assesses micronutrient intakes relative to Dietary Reference Intakes [9,10,11]. The objective of this study was to examine children’s dietary quality and micronutrient adequacy by food security in the household and among household children using a nationally representative sample of U.S. children from the National Health and Nutrition Examination Survey (NHANES) 2011–2014. Agreement between household food security and child food security was also examined.

## 2. Materials and Methods

### 2.1. Study Population

The NHANES is a nationally representative, cross-sectional survey that samples the noninstitutionalized, civilian residents of the United States using a complex, stratified, multistage probability cluster-sampling design [12]. The NHANES survey protocol was approved by the Research Ethics Review Board at the National Center for Health Statistics, and written informed consent was obtained for all participants or proxies. The NHANES protocol includes an in-home interview of demographics and self-reported health information, and a follow-up health measurement in a Mobile Examination Center for each participant. For survey participants who were under 16 years of age, a proxy provided information. We combined data from the 2011–2012 and 2013–2014 NHANES survey cycles, collected based on the four-year sample design that oversampled non-Hispanic non-Black Asian persons for the first time; these have most up-to-date dietary-supplement intake data because dietary-supplement use information is not yet available from the 2015–2016 cycle. From 2011, NHANES collected food-security information using the HFSSM alone and discontinued implementing several follow-up items on individual-level food security. The analytic sample included children ages 2–17 years with complete food-security information and reliable dietary recall data for at least one day (*n* = 5540). Children under 2 years were excluded, as Healthy Eating Index (HEI) score estimation is not possible in these ages because the DGA are for the U.S. population ages two and older.

### 2.2. Food-Security Assessment

The HFSSM was administered during the household interview, where an adult responded to the questions for the entire family [3]. The Household Food Security Scale was used to classify food security for households with children ages 17 years and younger, and the Child Food Security Scale was used to classify the experience of children in the household (i.e., household children). Based on the number of affirmative responses, household food security was categorized as high (0), marginal (1–2), low (3–7), or very low (8–18) [3]. Food security among household children was categorized as high (0), marginal (1), low (2–4), and very low (5–8) per NHANES documentation. Both household food security and food security among household children are reflective of conditions over the last 12 months, the reference period inherent to the HFSSM. 

### 2.3. Sociodemographic Variables

Sociodemographic characteristics that were linked with food-security status in the previous literature were examined, including individual characteristics (age, sex, race/Hispanic origin, and sibling status) and household characteristics (parental education level, family income, and food-assistance-program participation) [2,13]. Self-reported race/Hispanic origin groups as defined in the NHANES were non-Hispanic White, non-Hispanic Black, non-Hispanic Asian, Hispanic, and other races; the “other” race group was only included in the estimates for the total sample as recommended [12]. Household education level, defined as the education level of an adult household member who owns or rents the residence, was categorized as less than high school, high school or equivalent, some college or associate degree, and college graduate or above. The family poverty-to-income ratio (PIR) is the ratio of the annual family income to the poverty guideline established by the Department of Health and Human Services. PIR was categorized as <1, 1–1.3, 1.31–1.85, and >1.85. Families with PIR below 1 are considered “poor” by the Census Bureau [14]. PIR of 1.3 is an income eligibility criterion for the Supplemental Nutrition Assistance Program (SNAP), a federally funded food-assistance program that provides cash benefits for food [15,16]. A PIR of 1.85 also serves as the income eligibility criterion of other federal food-assistance programs such as the Special Supplemental Nutrition Program for Women, Infants, and Children (WIC) [16]. Current household SNAP participation status was categorized as participating, income-eligible but not participating, and income-ineligible and not participating. Lastly, whether a child is a singleton (i.e., an only child) or has one or more siblings was determined based on the information about the number of children in the household. When a child was living in a household with two or more children, the child was considered to have a sibling.

### 2.4. Dietary-Intake Data

Dietary-intake data were collected using the Automated Multiple-Pass Method as part of What We Eat in America using two 24-hour dietary recalls [17]. The first 24-hour recall was collected in person in the Mobile Examination Center, and the second recall was collected via phone 3 to 10 days later. During the 24-hour recall interview, information on the types and amounts of dietary supplements consumed during the 24-period prior to the interview was also collected, directly after the collection of food and beverage information. The USDA’s Food and Nutrient Database for Dietary Studies and NHANES Dietary Supplement Database were used to convert foods and beverages, and dietary supplements, respectively, to nutrient values.

Children’s dietary quality was characterized by the HEI 2015 because the HEI 2015 is based on the latest iteration of the DGA that reflects the most updated evidence on healthy eating. The HEI 2015 is a validated dietary-quality index that measures conformance to the 2015–2020 DGA [8]. The HEI 2015 rates densities of consumed food groups and nutrients rather than absolute amounts to evaluate dietary quality rather than dietary quantity. The HEI 2015 represents 13 dietary components with a total score of 100: adequacy components include total fruit (maximum score of 5), whole fruit (5), total vegetables (5), greens and beans (5), whole grains (10), dairy (10), total protein foods (5), seafood and plant proteins (5), and fatty acids (10), and moderation components include refined grains (10), sodium (10), added sugars (10), and saturated fats (10). The HEI 2015 components are similar with those of the HEI 2010 that assesses adherence to the 2010–2015 DGA, except that HEI 2015 includes separate components for ‘added sugars’ and ‘saturated fats’ instead of ‘empty calories’ component, does not include excessive energy from alcohol in any component, and allocates the legumes to all 4 components for vegetables and protein foods [8]. The scores were calculated at group level by the population ratio method based on first-day recalls [18,19] using publicly available SAS macros from the National Cancer Institute [20]. 

The MAR was chosen as an index of micronutrient adequacy. The MAR is calculated at an individual-person level based on nutrient intake from diet alone (i.e., dietary nutrient intake) or based on total nutrient intake from diet and dietary supplements [9,11]. Nutrient intake data were derived from the mean of two 24-hour dietary recalls when available, and from the first recall when only one reliable recall is available. Nutrient-adequacy ratio is the ratio of an individual’s nutrient intake to the Recommended Dietary Allowance (RDA) or Adequate Intakes (AI) from the Dietary Reference Intakes, truncated at 1.0 [21,22]. The MAR is the mean of NAR values for individual nutrients. This analysis included the NAR and MAR for the 9 shortfall micronutrients identified in the DGA: vitamins A, C, D, and E, folate, calcium, magnesium, iron, and potassium [23]. Vitamin A and E intakes from dietary supplements are not available in the 2011–2014 NHANES and are not included in this analysis. 

The prevalence of dietary-supplement use was estimated using information from a dietary-supplement questionnaire (DSQ) [24]. The DSQ was administered during the in-home interview in tandem with a home inventory, and collected information about any dietary-supplement use over the past 30 days to capture both habitual and episodic consumption of dietary supplements. If a child used any dietary supplement during the 30-day period, the child was classified as dietary-supplement user. As mentioned above, dietary-supplement-use information was also collected through 24-hour dietary recalls, and nutrient intake from dietary supplements was determined from 24-hour recall data.

### 2.5. Statistical Analysis

All statistical analyses were performed using SAS (version 9.4; SAS Institute, Inc., Cary, NC, USA) and SAS-callable SUDAAN (version 11; RTI International, Research Triangle Park, NC, USA) software. The 2011–2014 NHANES 4-year sample weights were used to account for differential probabilities of selection, nonresponse, and planned oversampling. Sociodemographic characteristics, including sex, age, PIR, race/Hispanic origin, household education level, sibling status, and SNAP participation status, were examined by household food security and food security among household children. The Satterthwaite-adjusted Wald Chi-square test was used to assess differences in the distribution of sociodemographic variables. Mean HEI 2015 score, dietary-supplement-use prevalence, and MAR from diet and from diet and dietary supplements were examined by household food security and food security among household children with pairwise *t* tests. Statistical significance was determined at a two-sided *p*-value <0.05.

To examine agreement between classifications of household food security and food security among household children, concordance (i.e., perfect agreement) was examined using the *n* obtained from Chi-square contingency tables. Raw Kappa agreement was estimated using the agree statement within *proc crosstab* using the survey design features. Additionally, “weighted” agreement was calculated using a Cichetti and Allison C-statistic for the four categories of food security, representing the relative proximity to each other [25]. The weighing of the agreement exerts more influence to observations closer to proximity rather than to perfect agreement alone, and is the preferred method to apply to scales that are ordinal in nature, whereas the unweighted Kappa is traditionally used for nominal scales [26]. The C-statistic is interpreted like a correlation coefficient and has been previously used to characterize nutritional indicators in the NHANES [27,28]. Statistical differences in C-statistics among sociodemographic subgroups were assessed by the overlap of confidence intervals because survey procedures do not exist for incorporating the NHANES sample weights and complex survey design features. The test of marginal homogeneity was used to test the null hypothesis that the probabilities of all the categories are the same, which would be expected based on random chance.

## 3. Results

In a representative sample of U.S. children in 2011–2014, both household food security and food security among household children were associated with children’s age, race/Hispanic origin, family income, household education level, and SNAP participation (Table 1). Compared to the high food-security category, children with marginal, low, and very low food security were more likely to be Non-Hispanic Black or Hispanic, and were living in families with lower incomes, lower educational attainment, and participating in SNAP. 

**Figure 1:**
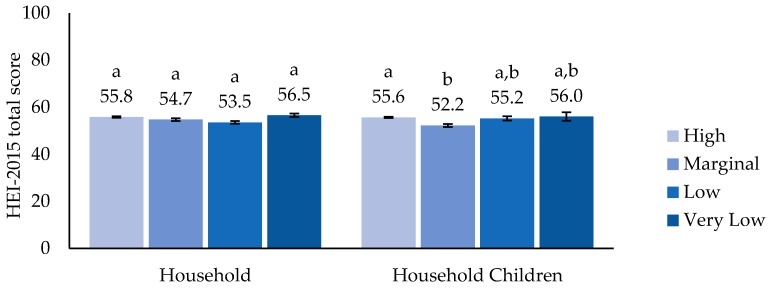
Healthy Eating Index (HEI) 2015 total score of U.S. children (2–17 years) by household food security and food security among household children. Estimates with different alphabet letters are significantly different based on pairwise t-tests within each classification at *p*-value < 0.05.

**Figure 2:**
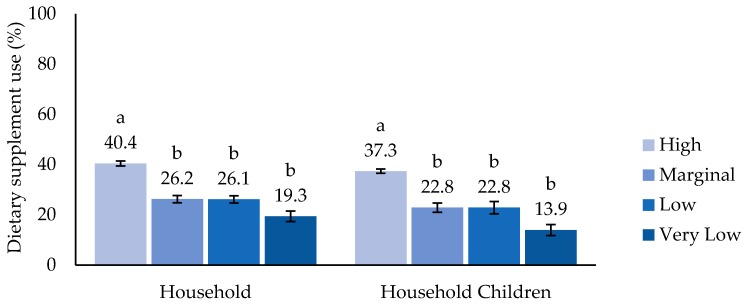
Prevalence of dietary-supplement use among U.S. children (2–17 years) by household food security and food security among household children. Estimates with different alphabet letters are significantly different based on pairwise t-tests within each classification at *p*-value < 0.05. Percentages may not sum to 100 owing to missing data and/or rounding.

**Figure 3:**
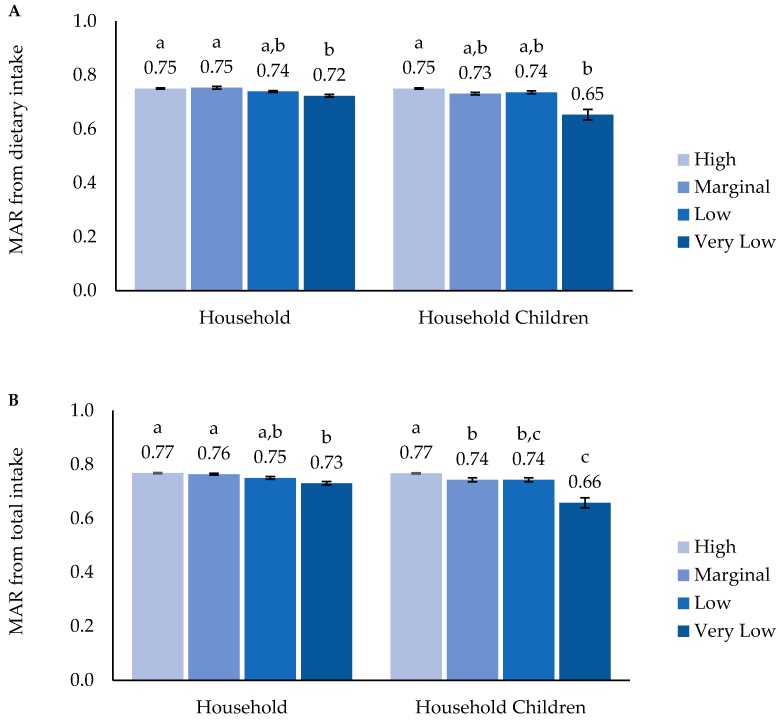
Mean Adequacy Ratio (MAR) from (**A**) dietary-nutrient intake and (**B**) total nutrient intake of U.S. children (2–17 years) by household food security and food security among household children. MAR was calculated from intakes of vitamins A, C, D, and E, folate, calcium, magnesium, iron, and potassium. Estimates with different alphabet letters are significantly different based on pairwise t-tests within each scale at *p*-value < 0.05.

There were no significant differences observed in children’s HEI 2015 scores by household food security; however, specifically among household children, HEI 2015 scores with marginal food security (52.2 ± SE 1.16) were lower compared to those with high food security (55.6 ± SE 0.62), but not different to other groups (Figure 1). Dietary-supplement use was highest in the high food security category, classified by both household food security (40.4%) and food security among household children (37.3%; Figure 2). The MAR calculated from diet alone was greater in the high food security category compared to the very low food-security category classed for both household and household children (Figure 3A). The MAR from total nutrient intake, inclusive of dietary supplements, was also higher in the high compared to the very low food-security category for both the household and among household children (Figure 3B). In addition, only when food security among children was classified was the MAR from total intake of the marginal food-security category lower than that of the high food-security category and higher than that of very low food-security category. These patterns largely remained after adjustment for age, race/Hispanic origin, family income, and household education level, except that a significant difference was observed in the MAR from diet alone between high and marginal food security among household children (data not shown).

Sixty-six percent of children 2–17 years were living in households with high food security, while 12%, 15%, and 7% were in households with marginal, low, and very low food security, respectively (Table 2). In contrast, 84% of children were living in situations where household children had high food security, followed by 6%, 8%, and 1% having marginal, low, and very low food security, respectively. Overall, 66% of observations were perfectly concordant and, among 28% of discordant observations, almost all were categorized into a higher food security category using the Child Food Security Scale compared to the Household Food Security Scale (data not shown). Based on the unweighted Kappa, fair agreement was observed (Kappa of 0.34) between household food security and food security among household children (data not shown) [29]. Analysis of the proximity of agreement in household and household children classifications using the C-statistic was 85%, and the test of marginal homogeneity suggested that the scales agreed beyond what was expected by chance (*p* < 0.0001). No substantial differences existed in the strength of agreement between boys and girls. When stratified by age group, the proximity of agreement was lower in the 2–5-year-olds (C-statistic = 0.84) than the 15–17-year-olds (C-statistic = 0.87). When stratified by race and Hispanic origin, agreement was highest in non-Hispanic Asians (C-statistic = 0.95), followed by non-Hispanic White (C-statistic = 0.89), non-Hispanic Black (C-statistic = 0.84), and Hispanics (C-statistic = 0.81). By family income, the agreement was lower in lower incomes (PIR ≤ 130%; C-statistic = 0.77) compared to higher incomes (PIR > 130%; C-statistic = 0.92); however, agreement did not vary based on singleton and sibling classification.

## 4. Discussion

Food insecurity can challenge a household’s ability to obtain food and make healthy choices, and may negatively influence dietary quality [23]. The unfavorable impact of food insecurity on childhood nutrition is especially concerning given the importance of this life stage for optimal growth and development, and the establishment of dietary behaviors that may persist into adulthood. Our findings suggest that both household food insecurity and food insecurity among household children are associated with lower micronutrient adequacy, as assessed by the MAR, and lower dietary-supplement use. A linear relationship was found only for food insecurity among household children in relationship with the MAR from total nutrient intake. Previous studies generally reported few differences in micronutrient intake from diet between food-secure (i.e., high and marginal food security) and food-insecure (i.e., low and very low food security) children, regardless of food-security scale [4,5]; however, some adverse associations of calcium and iron intakes with food insecurity among household children in older children were reported [30,31]. To the best of our knowledge, no study evaluated a summary measure derived from a group of nutrients (e.g., MAR) that reflects comprehensive nutrient intakes rather than single nutrients [7]. Furthermore, few studies have examined children’s total nutrient intake from both foods and dietary supplements by food-security status, although dietary-supplement use is known to differ by food security and household income [24]. Given that dietary supplements contribute substantial amounts of nutrients to children who use them [32], analysis of food-security comparisons should consider inclusion of nutrients from all sources [33].

The HEI 2015 score did not substantially differ by household food security or food security among children, consistent with many previous studies on food-based dietary-quality indices or food-group intakes [34,35,36,37,38]. There was very little variation in overall HEI scores, with all scores in the midrange of 50 out of a total possible 100 points (i.e., perfect adherence to the DGA). However, several other studies have reported lower fruit and vegetable intake, and higher added sugar intake in food-insecure children than in food-secure children, suggesting some possible constraints on specific dimensions of food intake [4,5]. It is notable that, in our analysis, the marginal food-security category had lower HEI scores than the high food-security category for classification by food security among children; the difference was largely driven by whole fruit, whole grain, and refined grain components (data not shown). Although the NHANES documentation identifies marginal food security among household children, the USDA has not separately reported the national prevalence estimates on this category due to a lack of expert consensus on language to describe it [39]. Given lower HEI 2015 scores and a lower MAR from total nutrient intakes inclusive of DS, marginal food security among household children may also pose a nutritional risk, although less severe, and should not be combined with full food security [40]. Further differences in children’s MAR across food-security categories were observed when classifying food security among household children compared to household food security. This suggests that food security among household children may be more sensitive to micronutrient adequacy, which was somewhat expected because the Child Food Security Scale was developed as a more specific classification of the experience of children compared with the Household Food Security Scale [6,39]. In contrast, two previous studies that conducted sensitivity analyses to compare the classification of household food security and food security among children observed fewer significant differences in micronutrient intake among Canadian children [41] and in food-group intake among U.S. children when using the Child Food Security Scale [36]. Inconsistencies with our findings could be due to dichotomization of food-security status, different dietary outcomes of interest (e.g., estimated usual intake of a single nutrient or food group), and different sample characteristics. 

There are many federal nutrition-assistance programs to mitigate food insecurity among children, for example, SNAP, the Special Supplemental Nutrition Program for WIC, National School Lunch and Breakfast Programs, and the Summer Food Service Program. While SNAP largely does not limit food choices, WIC, National School Lunch and Breakfast Programs, and Summer Food Service Program require offered foods to be aligned with the DGA. As children with less food security had lower micronutrient adequacy, continuous efforts to improve the nutrient quality of foods provided in these programs are warranted. In addition, more efforts to promote access to available programs and nutritious foods for food-insecure children not participating in federal programs may be needed. In this study, 32%, 18%, and 29% of children living in situations where household children had marginal, low, and very low food security, respectively, were not participating in SNAP. Moreover, 15%, 21%, and 6% of those with marginal, low, and very low food security among household children, respectively, were living in households that are income-ineligible for federal programs (i.e., PIR >1.85), although they may need nutrition assistance.

This analysis of NHANES data confirms the differences in the prevalence estimates of children living in food-insecure household (22.5%) and those with food insecurity among household children (9.7%), which has been reported by the U.S. Census data analysis [2,6]. In the current study, fewer children were categorized into the very low food-security category by the Child Food Security Scale compared to the Household Food Security Scale across all age groups. This is congruent with the work of Nord and Bickel [6] for younger children (2–5-year-olds), but different for older children; the previous work found more children to be categorized into “food insecurity with hunger among children” (now called very low food security among children) with the Child Food Security Scale than with the Household Food Security Scale in 6–14-year-olds and 15–17-year-olds. Household food security can differentially impact children when compared to adults within the same household, which is often explained as children being protected from the lack of food resources [42,43]. However, we cannot rule out potential bias in parental reporting on children’s experiences [44,45,46]. The strength of agreement between the two classification scales was lower in non-Hispanic Black and Hispanic children and those with lower family income; this may be partly due to higher food insecurity in these subgroups, but also highlights a need for special efforts in assessing food insecurity by race and Hispanic origin and family income. 

### Limitations and Strengths

This study uses cross-sectional data, so temporality could not be determined. In addition, the HFSSM captures chronic and episodic experiences of food insecurity over the past 12 months, while dietary recalls collect dietary intakes on one or two days that are 3–10 days apart. Thus, for those inconsistently experiencing food insecurity over time (e.g., summer vacation [47]), food-insecure experiences may not have been picked up in the recall time frame. However, the HEI 2015 scores were calculated using the population ratio method that provides a less biased estimate of the usual HEI score for a group of individuals compared to individual-person-level scores [18]. MAR calculation based on two dietary recalls may not reflect usual intakes [48], which would have affected the standard errors, but not the mean estimates; a method for usual intake estimation at individual level is not yet available [33]. All dietary data are subject to measurement errors, and it is possible that parents or caregivers reported intakes more favorably due to social-desirability bias [49], but little is known about the extent of reporting bias by food security. The sample size of the very low food-security group was very small and did not allow further stratification by age. Lastly, food-insecurity assessment is challenging. Neither household food security nor food security among children measures an individual child’s food insecurity, even though a child report can differ from an adult report [44,45,46]. Nonetheless, most large surveys and studies, including NHANES, interview an adult responsible for household food management about the food-security status of household members using the HFSSM. Nord and Hopwood [39] state that “standards have not yet been specified for the classification of individuals’ food-security status based on NHANES items”. Further efforts are needed to explore the best method to measure individual child food insecurity in national studies.

The strengths of this study include the use of a nationally representative sample of U.S. children and the estimation of the most updated HEI 2015 score. In addition, agreement between household food security and food security among household children classifications was assessed using the Cicchetti and Allison C-statistic that takes into consideration how far the categories are; for example, one category away (e.g., marginal food security in the household and low food security among household children) is considered a greater level of agreement than two or three categories away. The inclusion of nutrient intake from dietary supplements and parsing out singletons and children with siblings are novel contributions to the literature. 

## 5. Conclusions

In conclusion, micronutrient adequacy, but not dietary quality, of U.S. children differed significantly by food-security status classed by both household food insecurity and food insecurity among children. While agreement between household food security and food security of household children is very good, classification for food security among children appears to be more sensitive to dietary outcomes. The strength of agreement differed by children’s age and race/Hispanic origin, and family income. The findings of this study highlight the need for public health efforts to reduce food insecurity among U.S. children, and also serve to inform future studies of food-security scales in children.

## Figures and Tables

**Table 1:** Sociodemographic characteristics of U.S. children (2–17 years) by household food security and food security among household children ^1^.

	Household Food Security (*n* = 5540)					Food Security among Household Children (*n* = 5531)				
	High (*n* = 3196)	Marginal (*n* = 847)	Low (*n* = 1023)	Very Low (*n* = 474)	*p*-value ^2^	High (*n* = 4438)	Marginal (*n* = 425)	Low (*n* = 578)	Very Low (*n* = 90)	*p*-value ^2^
**Sex**										
Boy	50.8 (1.3)	48.9 (3.0)	50.1 (2.1)	55.6 (3.9)	0.49	50.2 (1.2)	50.2 (3.0)	57.3 (3.0)	46.7 (6.7)	0.14
Girl	49.2 (1.3)	51.1 (3.0)	49.9 (2.1)	44.4 (3.9)		49.2 (1.2)	49.8 (3.0)	42.7 (3.0)	53.3 (6.7)	
**Age**										
2–5 years	24.3 (1.0)	27.1 (2.3)	25.5 (1.8)	16.8 (2.3)	0.02	24.8 (0.9)	29.1 (3.4)	17.6 (2.4)	9.6 (2.9)	0.01
6–14 years	55.8 (1.7)	57.7 (2.9)	55.6 (2.0)	68.0 (3.6)		56.0 (1.6)	53.4 (3.0)	67.5 (2.9)	61.3 (5.8)	
15–17 years	20.0 (1.5)	15.2 (1.8)	19.0 (1.8)	15.2 (2.7)		19.2 (1.2)	17.4 (3.0)	15.0 (2.1)	29.0 (7.1)	
**Race and Hispanic origin**										
Non-Hispanic White	61.3 (3.5)	39.7 (4.9)	32.6 (5.6)	37.5 (6.2)	<0.001	55.8 (3.6)	37.1 (7.1)	34.3 (6.1)	32.5 (13.5)	<0.001
Non-Hispanic Black	10.9 (1.7)	20.5 (3.6)	20.5 (2.7)	22.4 (4.3)		12.5 (1.8)	25.5 (4.6)	23.7 (3.7)	21.6 (7.5)	
Non-Hispanic Asian	6.1 (0.6)	3.6 (1.0)	2.5 (1.0)	0.9 (0.4)		5.3 (0.5)	2.7 (0.8)	2.5 (1.1)	0.4 (0.4)	
Hispanic	17.6 (2.6)	29.7 (3.8)	40.2 (4.5)	32.7 (5.7)		22.0 (2.7)	29.4 (5.7)	33.4 (5.0)	41.9 (11.9)	
**Family Income**										
PIR <1	14.9 (1.8)	43.9 (4.1)	48.0 (3.6)	52.5 (4.8)	<0.001	22.2 (2.3)	48.2 (4.4)	47.8 (4.1)	48.0 (12.9)	<0.001
PIR 1–1.3	7.6 (1.1)	17.4 (3.2)	12.6 (2.3)	20.5 (4.6)		8.8 (1.0)	20.8 (3.6)	16.1 (3.6)	39.3 (14.0)	
PIR 1.31–1.85	10.5 (1.3)	12.4 (1.9)	16.6 (2.9)	13.9 (3.0)		11.3 (1.2)	16.2 (3.4)	15.5 (2.8)	6.9 (3.8)	
PIR >1.85	67.0 (2.5)	26.3 (5.4)	22.8 (3.9)	13.0 (4.7)		57.7 (3.0)	14.8 (2.8)	20.6 (4.9)	5.9 (5.6)	
**Household Education Level**										
Lower than high school	13.3 (1.6)	31.4 (4.3)	34.8 (3.6)	28.9 (3.7)	<0.001	18.4 (1.5)	27.8 (4.0)	27.7 (3.9)	35.3 (12.0)	<0.001
High school or equivalent	18.1 (1.8)	30.0 (2.8)	31.3 (3.7)	25.0 (3.6)		20.4 (1.7)	33.9 (5.6)	30.5 (4.5)	22.3 (9.5)	
Some college or associate degree	29.9 (2.0)	27.6 (3.7)	26.5 (3.4)	36.1 (4.0)		29.2 (1.7)	32.7 (5.6)	30.2 (3.6)	38.0 (11.8)	
College graduate or above	38.6 (2.4)	11.0 (2.8)	7.4 (1.6)	10.1 (4.3)		32.0 (2.3)	5.6 (2.4)	11.6 (4.2)	4.4 (2.5)	
**SNAP**										
Participating	16.8 (1.8)	45.9 (4.7)	49.4 (3.4)	54.9 (5.3)	<0.001	23.9 (2.3)	48.7 (5.3)	50.7 (5.0)	61.1 (12.1)	<0.001
Not participating, income-eligible	10.7 (1.3)	20.2 (4.0)	19.9 (2.3)	23.5 (3.3)		12.4 (1.3)	31.9 (3.5)	18.2 (2.7)	29.4 (10.6)	
Income-ineligible	72.5 (2.4)	33.9 (5.2)	30.6 (3.8)	21.6 (4.8)		63.7 (2.8)	19.4 (4.0)	31.2 (5.2)	9.5 (5.8)	

^1^ Values are % (SE). Percentages may not sum to 100 owing to missing data and/or rounding. ^2^ *p*-vales are from chi-square tests. PIR, family income-to-poverty ratio; SNAP, Supplemental Nutrition Assistance Program.

**Table 2:** Agreement between household food security and food security among household children among U.S. children (2–17 years) ^1^.

	Household Food Security % (SE)	Food Security among Household Children % (SE)	Concordance %	C-statistic (95% CI)	Heterogeneity Chi-Square^2^
**All**	*n* = 5540	*n* = 5531	66.0	0.851 (0.845, 0.857)	2413.2
High	65.5 (2.3)	84.4 (1.2)			
Marginal	12.0 (1.0)	6.0 (0.5)			
Low	15.2 (1.3)	8.4 (0.9)			
Very Low	7.3 (0.7)	1.3 (0.3)			
**Sex: Boys**	*n* = 2810	*n* = 2805	65.3	0.848 (0.839, 0.857)	1265.7
High	65.5 (2.5)	83.5 (1.4)			
Marginal	11.5 (1.1)	5.9 (0.6)			
Low	15.0 (1.4)	9.4 (1.1)			
Very Low	8.0 (1.0)	1.2 (0.2)			
**Sex: Girls**	*n* = 2730	*n* = 2726	66.7	0.854 (0.846, 0.863)	1148.8
High	65.5 (2.4)	85.3 (1.4)			
Marginal	12.5 (1.2)	6.0 (0.7)			
Low	15.4 (1.6)	7.3 (0.9)			
Very Low	6.6 (0.8)	1.4 (0.4)			
**Age Group: 2–5 years**	*n* = 1502	*n* = 1501	64.5	0.840 (0.828, 0.852)	696.8
High	65.6 (2.6)	86.3 (1.4)			
Marginal	13.4 (1.4)	7.2 (0.9)			
Low	16.0 (1.4)	6.1 (0.9)			
Very Low	5.0 (0.8)	0.5 (0.2)			
**Age Group: 6–14 years**	*n* = 3144	*n* = 3140	66.0	0.852 (0.844, 0.860)	1373.6
High	64.2 (2.6)	83.1 (1.6)			
Marginal	12.2 (1.1)	5.6 (0.6)			
Low	14.9 (1.6)	9.9 (1.3)			
Very Low	8.7 (1.0)	1.4 (0.3)			
**Age Group: 15–17 years**	*n* = 894	*n* = 890	68.6	0.867 (0.852, 0.881)	348.3
High	69.2 (3.1)	85.9 (2.0)			
Marginal	9.6 (1.6)	5.5 (1.1)			
Low	15.3 (1.9)	6.6 (1.2)			
Very Low	5.9 (1.3)	1.9 (0.8)			
**Race/Hispanic Origin: NH White**	*n* = 1336	*n* = 1334	73.5	0.885 (0.874, 0.896)	423.5
High	76.3 (2.2)	89.6 (1.5)			
Marginal	9.0 (1.4)	4.2 (0.7)			
Low	9.4 (1.4)	5.5 (1.1)			
Very Low	5.2 (1.0)	0.8 (0.4)			
**Race/Hispanic Origin: NH Black**	*n* = 1523	*n* = 1520	60.8	0.836 (0.825, 0.847)	802.4
High	49.8 (2.8)	73.7 (2.3)			
Marginal	17.1 (1.5)	10.6 (1.6)			
Low	21.7 (2.5)	13.8 (1.7)			
Very Low	11.4 (1.5)	1.9 (0.7)			
**Race/Hispanic Origin: NH Asian**	*n* = 572	*n* = 571	85.8	0.946 (0.935, 0.958)	77.6
High	81.9 (3.8)	92.4 (1.9)			
Marginal	8.9 (1.9)	3.3 (1.0)			
Low	7.9 (2.8)	4.2 (1.8)			
Very Low	1.3 (0.5)	0.1 (0.1)			
**Race/Hispanic Origin: Hispanic**	*n* = 1776	*n* = 1774	57.9	0.805(0.793, 0.817)	1086.8
High	48.9 (3.6)	78.5 (2.3)			
Marginal	15.1 (1.9)	7.4 (1.3)			
Low	25.9 (2.3)	11.8 (1.4)			
Very Low	10.1 (1.6)	2.2 (0.7)			
**Family Income: PIR ≤1.3**	*n* = 2494	*n* = 2489	48.7	0.774(0.764, 0.784)	2090.3
High	40.2 (2.9)	71.3 (2.1)			
Marginal	19.8 (1.8)	10.7 (0.9)			
Low	25.1 (1.9)	15.1 (1.6)			
Very Low	15.0 (1.4)	2.9 (0.7)			
**Family Income: PIR >1.3**	*n* = 2710	*n* = 2707	81.1	0.917 (0.911, 0.925)	558.3
High	80.1 (1.7)	92.0 (1.0)			
Marginal	7.2 (1.1)	2.8 (0.4)			
Low	9.4 (1.2)	4.9 (0.9)			
Very Low	3.2 (0.7)	0.2 (0.1)			
**Sibling: Only Child**	*n* = 1046	*n* = 1040	70.1	0.863 (0.848, 0.877)	369.2
High	71.0 (2.0)	90.5 (1.0)			
Marginal	11.1 (1.2)	4.3 (0.7)			
Low	13.0 (1.4)	4.5 (0.8)			
Very Low	4.9 (0.8)	0.6 (0.2)			
**Sibling: Has Sibling**	*n* = 4494	*n* = 4491	65.0	0.848 (0.842, 0.855)	2054.6
High	64.1 (2.6)	82.9 (1.4)			
Marginal	12.2 (1.2)	6.4 (0.5)			
Low	15.8 (1.5)	9.3 (1.1)			
Very Low	7.9 (0.8)	1.4 (0.3)			

^1^ Percentages may not sum to 100 owing to missing data. Concordance indicates the percentage of concordant observations. C-statistic and heterogeneity are from a weighted kappa approach proposed by Cicchetti and Allison [25]. Concordance, C-statistic, and heterogeneity do not account for the National Health and Nutrition Examination Survey (NHANES) survey design features or sampling weights, but all other values are survey weighted. NH, Non-Hispanic. ^2^ All *P*-values were below 0.0001.

## References

[1] Anderson S.A. (1990). Core Indicators of Nutritional State for Difficult-to-Sample Populations. J. Nutr..

[2] Coleman-Jensen A., Rabbit M.P., Gregory C.A., Singh A. (2018). Household Food Security in the United States in 2017.

[3] Bickel G., Nord M., Price C., Hamilton W., Cook J. (2000). Guide to Measuring Household Food Security.

[4] Eicher-Miller H.A., Zhao Y. (2018). Evidence for the age-specific relationship of food insecurity and key dietary outcomes among US children and adolescents. Nutr. Rev..

[5] Hanson K.L., Connor L.M. (2014). Food insecurity and dietary quality in US adults and children: a systematic review. Am. J. Clin. Nutr..

[6] Nord M., Bickel G. (2002). Measuring Children’s Food Security in U.S. Households, 1995–1999.

[7] Kant A.K. (1996). Indexes of Overall Diet Quality: A Review. J. Am. Diet. Assoc..

[8] Krebs-Smith S.M., Pannucci T.E., Subar A.F., Kirkpatrick S.I., Lerman J.L., Tooze J.A., Wilson M.M., Reedy J. (2018). Update of the Healthy Eating Index: HEI-2015. J. Acad. Nutr. Diet..

[9] Guthrie H.A., Scheer J.C. (1981). Validity of a Dietary Score for Assessing Nutrient Adequacy. J. Am. Diet. Assoc..

[10] Raffensperger S., Kuczmarski M.F., Hotchkiss L., Cotugna N., Evans M.K., Zonderman A.B. (2010). Effect of Race and Predictors of Socioeconomic Status on Diet Quality in the HANDLS Study Sample. J. Med. Assoc..

[11] Kuczmarski M.F., Beydoun M.A., Shupe E.S., Pohlig R.T., Zonderman A.B., Evans M.K. (2017). Use of Dietary Supplements Improved Diet Quality But Not Cardiovascular and Nutritional Biomarkers in Socioeconomically Diverse African American and White Adults. J. Nutr. Gerontol. Geriatr..

[12] National Center for Health Statistic (2013). National Health and Nutrition Examination Survey: Analytic Guidelines, 2011–2012.

[13] Nord M. (2009). Food Insecurity in Households with Children: Prevalence, Severity, and Household Characteristics.

[14] U.S. Department of Health and Human Services Poverty Guidelines. https://aspe.hhs.gov/poverty-guidelines.

[15] Gundersen C. (2013). Food Insecurity Is an Ongoing National Concern123. Adv. Nutr. Int. J..

[16] Oliveira V. (2018). The Food Assistnce Landscape: Fy 2017 Annual Report.

[17] Blanton C.A., Moshfegh A.J., Kretsch M.J., Baer D.J. (2006). The USDA Automated Multiple-Pass Method Accurately Estimates Group Total Energy and Nutrient Intake. J. Nutr..

[18] Freedman L.S., Guenther P.M., Krebs-Smith S.M., Kott P.S. (2008). A Population’s Mean Healthy Eating Index-2005 Scores Are Best Estimated by the Score of the Population Ratio when One 24-Hour Recall Is Available. J. Nutr..

[19] Kirkpatrick S.I., Reedy J., Krebs-Smith S.M., Pannucci T.E., Subar A.F., Wilson M.M., Lerman J.L., Tooze J.A. (2018). Applications of the Healthy Eating Index for Surveillance, Epidemiology, and Intervention Research: Considerations and Caveats. J. Acad. Nutr. Diet..

[20] National Cancer Institute The Healthy Eating Index: Sas Code. https://epi.grants.cancer.gov/hei/sas-code.html.

[21] Marshall T.A., Gilmore J.M.E., Broffitt B., Stumbo P.J., Levy S.M. (2005). Diet Quality in Young Children Is Influenced by Beverage Consumption. J. Am. Nutr..

[22] Institute of Medicine Food Nutrition Board (2006). Dietary Reference Intakes: The Essential Guide to Nutrient Requirements.

[23] U.S. Department of Health Human Services (2015). 2015–2020 Dietary Guidelines for Americans.

[24] Jun S., Cowan A.E., Tooze J.A., Gahche J.J., Dwyer J.T., Eicher-Miller H.A., Bhadra A., Guenther P.M., Potischman N., Dodd K.W. (2018). Dietary Supplement Use among U.S. Children by Family Income, Food Security Level, and Nutrition Assistance Program Participation Status in 2011–2014. Nutrients.

[25] Cicchetti D.V., Allison T. (1971). A New Procedure for Assessing Reliability of Scoring EEG Sleep Recordings. Am. J. EEG Technol..

[26] Vanbelle S. (2016). A New Interpretation of the Weighted Kappa Coefficients. Psychometrika.

[27] Bailey R.L., Fulgoni V.L., Taylor C.L., Pfeiffer C.M., Thuppal S.V., McCabe G.P., A Yetley E. (2017). Correspondence of folate dietary intake and biomarker data123. Am. J. Clin. Nutr..

[28] Dye B.A., Barker L.K., Selwitz R.H., Lewis B.G., Wu T., Fryar C.D., Ostchega Y., Beltran E.D., Ley E. (2007). Overview and quality assurance for the National Health and Nutrition Examination Survey (NHANES) oral health component, 1999–2002. Community Dent. Oral Epidemiol..

[29] Landis J.R., Koch G.G. (1977). The measurement of observer agreement for categorical data. Biometrics.

[30] Eicher-Miller H.A., Mason A.C., Weaver C.M., McCabe G.P., Boushey C.J. (2009). Food Insecurity Is Associated with Iron Deficiency Anemia in Us Adolescents. Am. J. Clin. Nutr..

[31] Eicher-Miller H.A., Mason A.C., Weaver C.M., McCabe G.P., Boushey C.J. (2011). Food Insecurity Is Associated with Diet and Bone Mass Disparities in Early Adolescent Males but Not Females in the United States. J. Nutr..

[32] Bailey R.L., Fulgoni V.L., Keast D.R., Lentino C.V., Dwyer J.T. (2012). Do Dietary Supplements Improve Micronutrient Sufficiency in Children and Adolescents?. J. Pediatr..

[33] Bailey R.L., Dodd K.W., Gahche J.J., Dwyer J.T., E Cowan A., Jun S., Eicher-Miller H.A., Guenther P.M., Bhadra A., Thomas P.R. (2019). Best Practices for Dietary Supplement Assessment and Estimation of Total Usual Nutrient Intakes in Population-Level Research and Monitoring. J. Nutr..

[34] Knol L.L., Haughton B., Fitzhugh E.C. (2004). Food insufficiency is not related to the overall variety of foods consumed by young children in low-income families. J. Am. Diet. Assoc..

[35] Bhattacharya J., Currie J., Haider S. (2004). Poverty, food insecurity, and nutritional outcomes in children and adults. J. Heal. Econ..

[36] Rossen L.M., Kobernik E.K. (2016). Food Insecurity and Dietary Intake among Us Youth, 2007–2010. Pediatr. Obes..

[37] Trapp C.M., Burke G., Gorin A.A., Wiley J.F., Hernandez D., Crowell R.E., Grant A., Beaulieu A., Cloutier M.M. (2015). The Relationship between Dietary Patterns, Body Mass Index Percentile, and Household Food Security in Young Urban Children. Child. Obes..

[38] Jackson J.A., Smit E., Manore M.M., John D., Gunter K. (2015). The Family-Home Nutrition Environment and Dietary Intake in Rural Children. Nutrients.

[39] Nord M., Hopwood H. (2007). Recent Advances Provide Improved Tools for Measuring Children’s Food Security. J. Nutr..

[40] Cook J.T., Black M., Chilton M., Cutts D., Ettinger de Cuba S., Heeren T.C., Rose-Jacobs R., Sandel M., Casey P.H., Coleman S. (2013). Are Food Insecurity’s Health Impacts Underestimated in the U.S. Population? Marginal Food Security Also Predicts Adverse Health Outcomes in Young U.S. Children and Mothers. Adv. Nutr..

[41] Kirkpatrick S.I., Tarasuk V. (2008). Food Insecurity Is Associated with Nutrient Inadequacies among Canadian Adults and Adolescents. J. Nutr..

[42] Hamilton W.L., Cook J.T., Thompson W.W., Buron L.F., Frongillo E.A., Olson C.M., Wehler C.A. (1997). Household Food Security in the United States in 1995: Summary Report of the Food Security Measurement Project.

[43] McIntyre L., Glanville N.T., Raine K.D., Dayle J.B., Anderson B., Battaglia N. (2003). Do low-income lone mothers compromise their nutrition to feed their children?. Can. Med. Assoc. J..

[44] Fram M.S., Frongillo E.A., Jones S.J., Williams R.C., Burke M.P., Deloach K.P., Blake C.E. (2011). Children Are Aware of Food Insecurity and Take Responsibility for Managing Food Resources. J. Nutr..

[45] Chavez F.L.C., Hernandez D.C., Harris G.J., Grzywacz J.G. (2017). Household Food Security Discordance Among Latino Adolescents and Parents. Am. J. Health Behav..

[46] Nalty C.C., Sharkey J.R., Dean W.R. (2013). Children’s reporting of food insecurity in predominately food insecure households in Texas border colonias. Nutr. J..

[47] Nord M., Romig K. (2006). Hunger in the Summer. J. Child. Poverty.

[48] Dodd K.W., Guenther P.M., Freedman L.S., Subar A.F., Kipnis V., Midthune D., Tooze J.A., Krebs-Smith S.M. (2006). Statistical Methods for Estimating Usual Intake of Nutrients and Foods: A Review of the Theory. J. Am. Diet. Assoc..

[49] Subar A.F., Freedman L.S., A Tooze J., I Kirkpatrick S., Boushey C., Neuhouser M.L., E Thompson F., Potischman N., Guenther P.M., Tarasuk V. (2015). Addressing Current Criticism Regarding the Value of Self-Report Dietary Data12. J. Nutr..

